# The radial nerve bridging plate technique for humeral shaft fractures: an approach facilitating plate removal without impeding fracture union - a case series

**DOI:** 10.1007/s00068-026-03334-x

**Published:** 2026-09-23

**Authors:** Guy Putzeys, Leendert H. T. Nugteren, Peter Reynders-Frederix, Dennis Den Hartog

**Affiliations:** 1 Orthopedic and Trauma Department, AZ Groeninge Hospital, Kortrijk, Belgium; 2 Trauma Research Unit, Department of Surgery, Erasmus MC, University Medical Center Rotterdam, P.O. Box 2040, Rotterdam, 3000 CA The Netherlands; 3 Orthopedic Department, CHU Brussels, Brussels, Belgium

**Keywords:** Humeral shaft fracture, Removal of humerus plate, Radial nerve bridging, Radial nerve palsy

## Abstract

**Background:**

Removing a plate of the humeral shaft with the radial nerve on top of the plate is challenging and carries a high risk for iatrogenic radial nerve palsy (iRNP). This study aimed to assess the incidence of iRNPs following plate removal of a radial nerve bridging plate (RNBP) and to evaluate outcomes of this RNBP in treatment of a humeral shaft fracture.

**Methods:**

This was a single-center retrospective case series. Patients treated between May 2013 and December 2025 with a RNBP for a humeral shaft fracture or nonunion were included. The primary outcome measure was the incidence of iRNP after removal of the RNBP. Secondary outcome measures were the incidence of iRNP, malunion and nonunion after primary surgery.

**Results:**

Of 48 included patients, the mean follow-up duration was 24 months. In 11 patients the plate was removed, no iRNP occurred in these patients. Of 41 patients who had no primary RNP after the fracture, seven patients experienced a temporary iRNP after primary surgery. All of the RNPs fully recovered with a mean recovery time of 12.3 weeks (range: 1–32). There were three nonunions, one of which was a pathological fracture, and one was a nonunion of a surgical neck fracture in a multilevel fracture.

**Conclusion:**

The RNBP technique allows safe and uneventful removal of the RNBP without iRNP, while maintaining the efficacy of plate fixation to treat humeral shaft fractures. It facilitates the decision to remove an implant if it seems indicated without risk of iatrogenic nerve damage.

## Introduction

Humeral shaft fractures represent 1–3% of all fractures with an incidence rate of 14.5 per 100,000 person-years [1]. There is growing evidence that surgical treatment lowers the risk of malunion, non-union, and need for revision surgery, while also enabling a quicker return to daily activities [2, 3]. Common surgical techniques are intramedullary nailing or plate fixation, the latter open or minimal invasive (MIPO). A recently published large prospective cohort study described that plate fixation is associated with faster functional recovery, higher union rates, and fewer implant-related complications and surgical reinterventions compared with intramedullary nailing [4].

Iatrogenic radial nerve palsy (iRNP) occurs more frequently after open plate fixation of acute humeral shaft fractures compared with intramedullary nailing or MIPO [5]. Most iRNP recover spontaneously, with spontaneous recovery of RNP in 89%-99% of cases [6, 7].

The plate is traditionally positioned beneath the radial nerve, which makes later removal hazardous, since it can be difficult to identify and dissect the radial nerve from surrounding adhesions and scar tissue [8]. A meta-analysis reported a re-intervention rate of 12.6% after open plate fixation [6]. The incidence of RNP after plate removal remains unclear. Rates of iRNP after secondary surgery can only be cautiously inferred from papers on humeral nonunion surgery, typically involving a mixed caseload of primary fracture treatment, using different implants and approaches. Furthermore, details on the need for radial nerve exploration and whether the radial nerve is in the field of the plate are lacking in these studies. In a study by Kakazu et al. on humeral nonunions treated with a plate, 20% of cases primarily treated with plate fixation developed a radial nerve palsy after surgical nonunion treatment [9]. In a multicenter retrospective study of 379 patients with retained radial nerve function who were treated operatively for a humeral shaft nonunion, 26 patients (6.9%) had an iRNP, of whom three patients experienced persistent palsy [10]. In a systematic review including three studies with 105 cases of humeral nonunions treated with plate osteosynthesis, the rate of iRNP was 15% (9/60) for the anterolateral approach, 17% (2/12) for the lateral approach and 18% (6/33) for the posterior approach [11].

Avoiding positioning the radial nerve on top of a plate that crosses the nerve during primary surgery should allow for safer plate removal during secondary surgery. Trans-fracture transposition of the radial nerve during plate surgery is one way to achieve this. In a study of 19 cases of trans-fracture transposition of the radial nerve, six patients underwent plate removal with no secondary RNP [8]. However, this method is rarely reported in the literature and does not appear to have been widely adopted. As an alternative, the radial nerve can be protected using a radial nerve bridging plate (RNBP), a locally bent plate to bridge the nerve during the initial surgery. The primary aim of this study was to assess the number of iRNP after plate removal of a RNBP in patients with a humeral shaft fracture or nonunion. Secondary aims were to assess the efficacy of the RNBP technique in terms of union rates and complications. It was hypothesized that the RNBP technique is not associated with iRNP after plate removal, while maintaining the effectiveness of plate fixation in primary treatment.

## Methods

### Study design and setting

All patients treated for a humeral shaft fracture or nonunion with the RNBP technique between May 2013 and December 2025 were included in this retrospective case series. The RNBP technique was introduced in 2013 and was used for all patients whenever the plate crossed the radial nerve. Patients were retrieved from a prospectively maintained database. Treatment was performed at a Level I trauma center by a single consultant orthopedic surgeon with more than 25 years of experience in the surgical management of humeral fractures and serving as faculty at several international upper-extremity educational courses. Data were retrospectively collected from the electronic medical records. The study was approved by the ethical committee of AZ Groeninge, Kortrijk (B3962024000071).

### Participants

All patients aged 16 years or older with a displaced (peri-implant) humeral shaft fracture, with or without extension to the proximal or distal humerus, or a humeral shaft nonunion, treated with RNBP osteosynthesis in the study period were eligible for inclusion. One exclusion criterion was a follow-up less than six months.

### Data collection and outcome measures

The medical records and radiographs of all eligible patients were reviewed, documenting demographic data and complications. The primary outcome was the number of iRNPs after removal of the RNBP. Secondary outcomes were the incidence of iRNP, malunion and nonunion after primary RNBP osteosynthesis. Patients with preoperative radial nerve injury (motor or sensory) were not diagnosed with iRNP. The fracture type and location were classified according to the AO/OTA classification [12]. Fracture location was divided into the proximal, middle, and distal thirds of the humeral shaft. In addition, combined fractures of the shaft and proximal or distal humerus were identified as multifocal humeral shaft fractures. The fracture position relative to the wave segment was categorized as proximal to, at-the-level of, or distal to the wave segment. In all cases a preoperative X-ray was available, in 11 out of 48 cases the X-ray was supplemented with a computed tomography (CT) scan. Nonunion was defined as absence of bridging callus in ≥ 3 of 4 cortices by 6 months [13]. Malunion was defined as radiographic evidence of angulation 20° in any plane [14, 15]. A RNP was defined as weakness or paralysis of wrist, finger, and thumb extension.

### Surgical technique

**Fig. 1:**
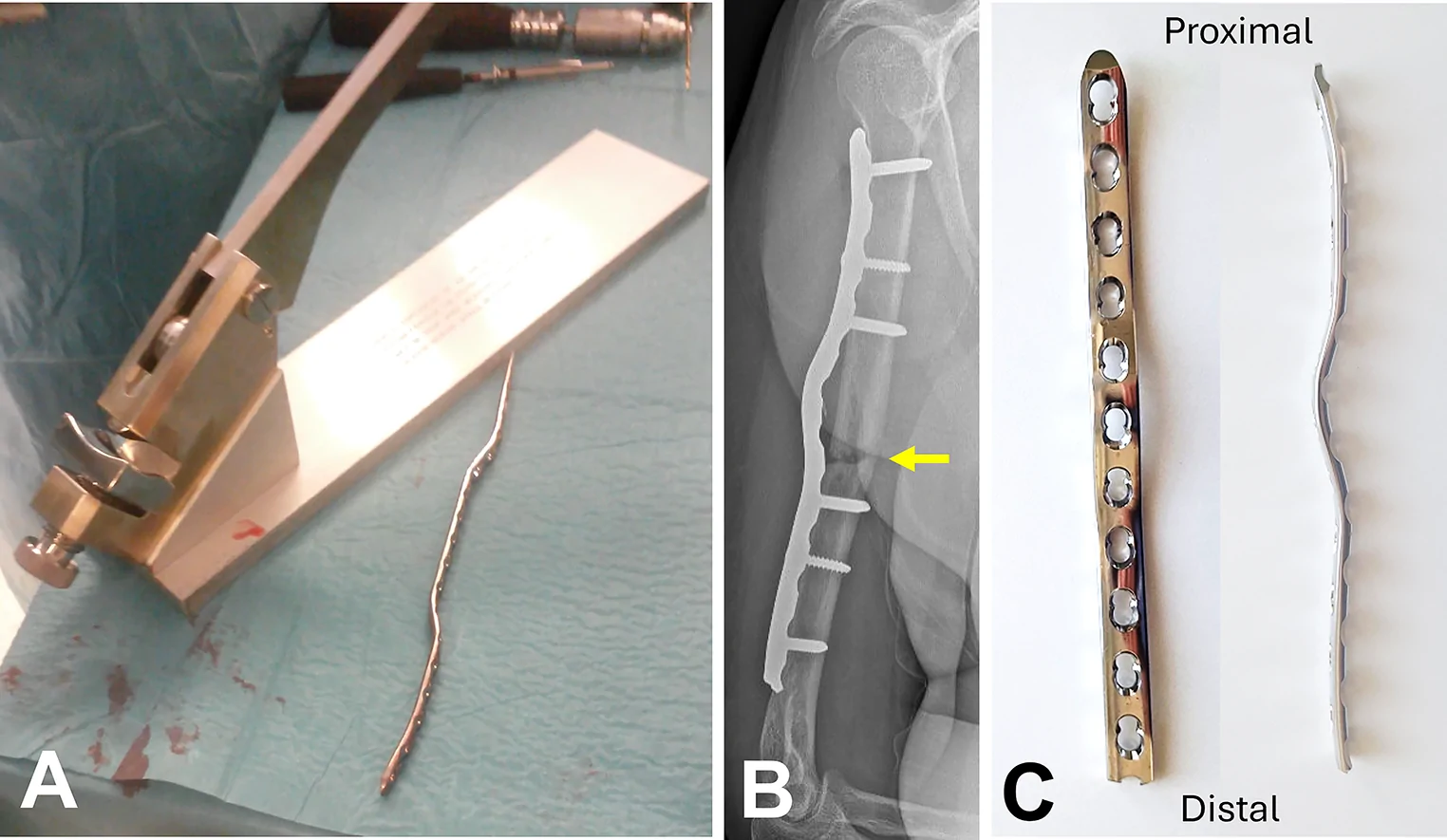
**A**: Intraoperative view of a plate bender. Next to it a shaped stainless steel metaphyseal plate with a long wave in the middle. **B**: Postoperative Xray of a humerus showing a posteriorly applied RNBP with a short wave for a fracture in the middle one-third of the humerus at the level of the wave segment (yellow arrow). **C**: Same plate after removal

**Fig. 2:**
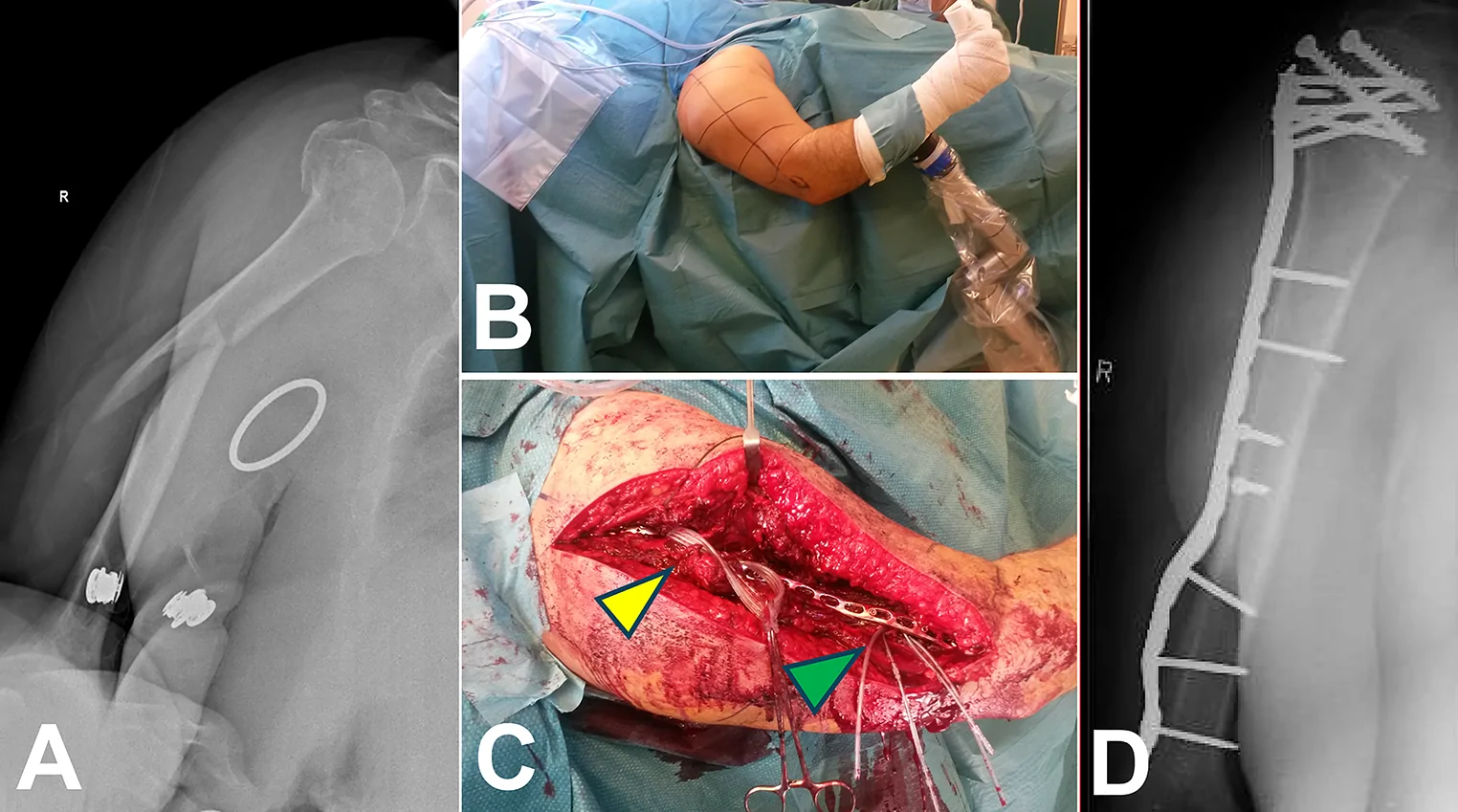
**A**: Preoperative x-ray of a spiral humeral shaft fracture in combination with a displaced head split fracture of the proximal humerus in a 67-year-old male. **B**: Intraoperative view positioning of the patient for a lateral approach. **C**: Intraoperative view of the lateral approach with a deltoid split and isolation of a deltoid muscle bridge containing the axillary nerve (yellow triangle) and a lateral positioned RNBP with the radial nerve and a side branch running below the wave segment of the plate (green triangle). **D**: Postoperative view one month after surgery

**Fig. 3:**
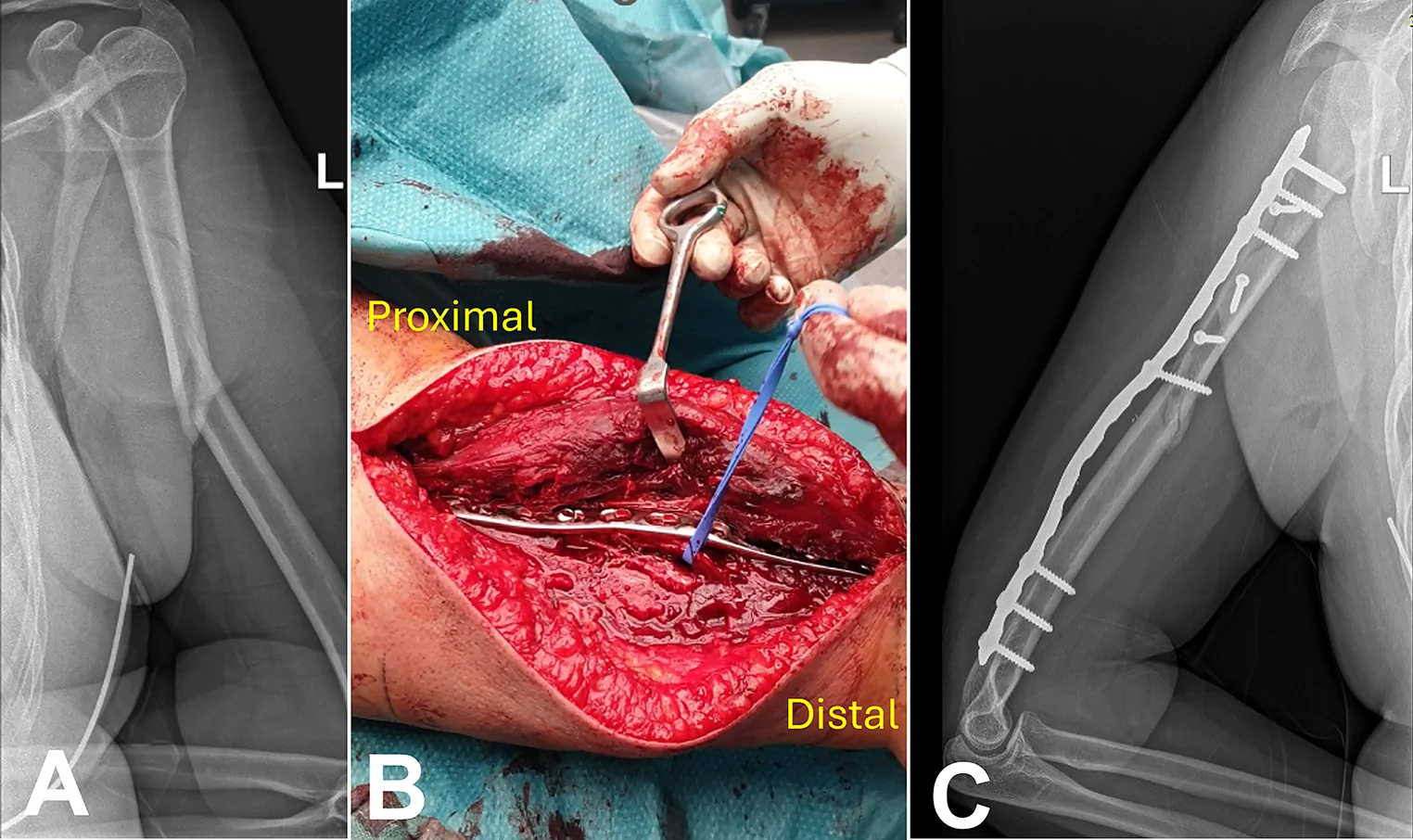
**A**: Preoperative x-ray of a spiral midshaft humerus fracture in a 50-year-old female. **B**: Intraoperative view of a posterior RNBP with the looped radial nerve underneath the plate at the wave segment. **C**: One month postoperative lateral radiograph view

**Fig. 4:**
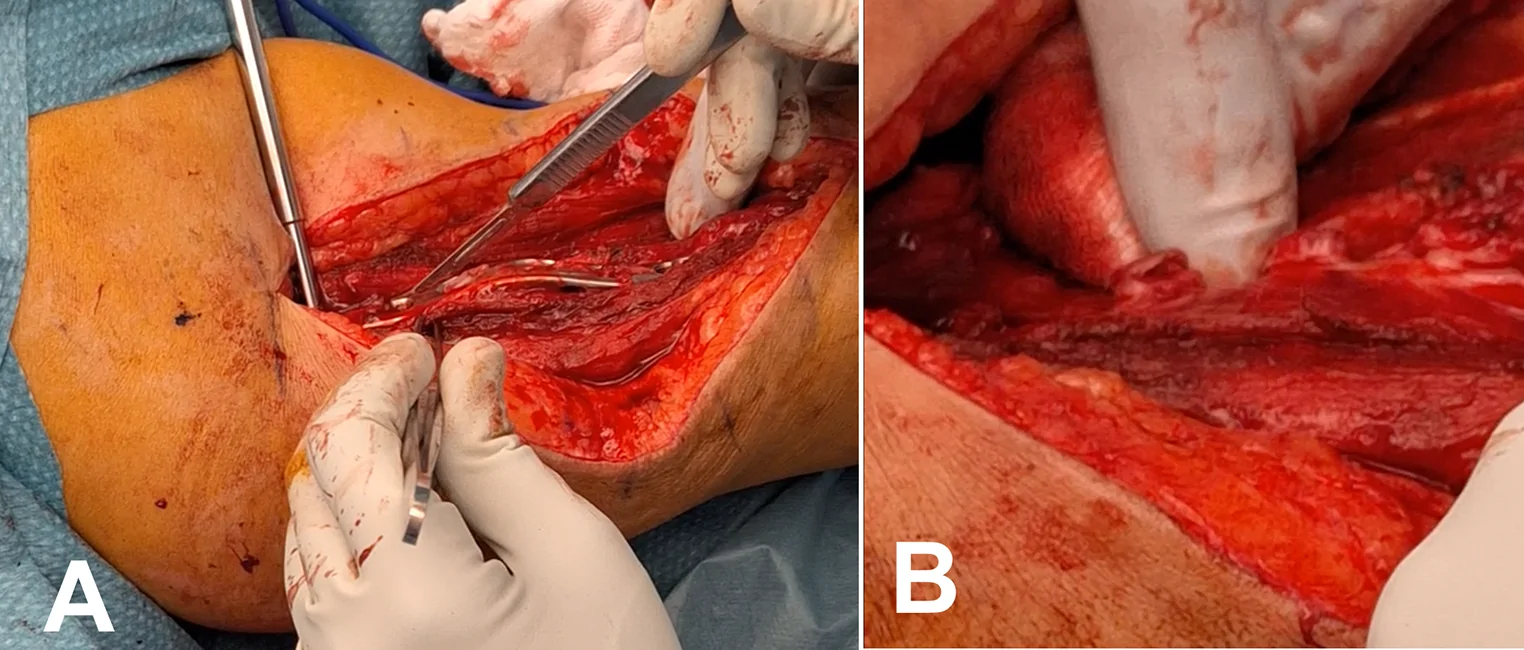
**A**: Intraoperative view of the clearance of a posterior RNBP with a sharp knife at the wave segment of the plate. **B**: Gentle palpation of the radial nerve in the scar tissue after removal of the RNB plate with scar papules where the wave segment was located

In each case, the radial nerve was identified and mobilized. At the surgeon’s discretion, standard anatomical reduction with compression when possible or else a bridging technique was applied. All plates were fixated with at least three bicortical screws at each side of the fracture with the largest distance possible between them. In case of periprosthetic fractures, cable wires were used at the level of the prosthesis stem. Except for one case, in which a titanium 14 shaft hole helical plate from the A.L.P.S proximal humeral plating system was used, all plates used were stainless steel locking plates. If deemed necessary, a small semi tubular plate was used to assist reduction of the fracture. The RNBP has a wave-like curve bent into the portion of the straight plate corresponding to the radial nerve trajectory crossing the plate. The curve was created intraoperatively using a four-step technique with a manually operated plate bender, as described earlier (Fig. 1) [16]. Lateral plating was typically chosen in fractures of the proximal and middle third of the shaft [17]. The straight plate was bent distally into a short, trapezoidal bend measuring about 4 cm in length to bridge the radial nerve. Additionally, the distal end of the plate was bent into a smooth curve that fit the curve of the lateral column of the distal humerus (Fig. 2). The case shown in Fig. 2 was treated at a time when helical plates were not yet available in the study center, and a long, straight lateral plate was therefore selected. Since May 2017, the lateral RNBP technique has been fully replaced by the helical plate, which, due to its anterior position distally, eliminates the need to bridge the radial nerve. Posterior plating was chosen in fractures of the middle and/or distal third of the shaft. Both a posterior triceps-splitting and a posterior triceps-sparing approach were used. After splitting the triceps, the radial nerve was identified distally into the zone of injury and proximally into the spiral groove to ensure mobility. The straight plate was bent proximally into a long, trapezoidal bend measuring about 6–9 cm to bridge the radial nerve, which typically courses over a longer segment of bone proximally than it does distally lateral side (Fig. 3). In the case presented in Fig. 3 a posterior approach was preferred over an anterolateral approach to facilitate radial nerve protection during fracture reduction. Postoperative management consisted of a removable sling for 4-6 weeks and early mobilization. Physiotherapy started at the earliest 4 weeks postoperatively. Postoperative radiographic assessment was performed on X-rays obtained immediately following surgery and on the most recent X-ray available. Since in all patients the radial nerve was explored during primary surgery, no secondary exploration was performed in case of iRNP. If plate removal indicated, the incision was made directly from the skin to the plate without exploring the radial nerve (Fig. 4). To avoid irritation and palsy of the radial nerve, cauterization should not be used at the level of the wave.

### Statistics

Descriptive statistics were presented. For continuous data, the mean and the range were reported. The number and frequencies were reported for categorical data. Due to the sample size and study design, the analyses were descriptive. Since no comparisons were made between groups, no formal hypothesis testing was conducted. Missing values were not imputed.

## Results

### Baseline characteristics

Baseline characteristics are presented in Table 1. During the study period, 52 patients were treated with the RNBP technique. Four patients had insufficient follow-up, thus 48 patients, with a mean age of 63 years (range: 17–93), were included. Twenty-eight patients (58%) were female. Twenty-two were left-sided and 26 were right-sided. The mean follow-up duration was 24 months (range: 6–84). Except for four patients with additional fractures at other locations, all fractures were isolated to the humerus.

**Table 1:** Patient and fracture characteristics

		RNBP
	*N*	(*n* = 48)
**Patient characteristics**		
Age at time of surgery (years)	48	63 (17–93)
Sex (female)	48	28 (58%)
Follow up (months)	48	24 (6–83)
**Fracture characteristics**		
Fracture location	48	
Proximal third		11 (23%)
Middle third		22 (46%)
Distal third		15 (31%)
AO-OTA classification	48	
12A1		17 (35%)
12A2		5 (10%)
12A3		5 (10%)
12B2		14 (29%)
12B3		1 (2%)
12C2		2 (4%)
12C3		4 (8%)
Open fracture	48	2 (4%)
Primary RNP	48	7 (15%)
**Surgical details**		
Surgery indication	48	
Primary fracture		34 (71%)
Peri-implant fracture		6 (13%)
Pathological fracture		4 (8%)
Nonunion		1 (2%)
Early osteosynthesis failure		3 (6%)
Approach	48	
Lateral		21 (44%)
Posterior		27 (56%)
Surgery duration (minutes)	46	116 (49–203)
Wave segment location	48	
Proximal to fracture		6 (13%)^1^
Same level as fracture		26 (54%)^2^
Distal to fracture		16 (33%)^3^
Technique	48	
Compression		35 (73%)
Bridging		10 (21%)
Both		3 (6%)

Data are presented as mean (range) or N (%)

N* represents the number of cases with available data

^1^All of these plates were positioned posteriorly

^2^Five plates were positioned laterally, 21 posteriorly

^3^All of these plates were positioned laterally

RNBP, radial nerve bridging plate; RNP, radial nerve palsy

### Fracture characteristics

Fracture characteristics are presented in Table 1. Of the fractures, 11 (23%) occurred in the proximal third, 22 (46%) in the middle third, and 15 (31%) in the distal third of the humerus. There were 36 fractures (75%) limited to the shaft and 12 multilevel fractures (25%), 10 of which involved the proximal humerus and two the distal humerus. According to the AO/OTA classification, 27 fractures (56%) were classified as type 12 A, 15 (31%) as type 12B, and 6 (13%) as type 12 C. Two fractures were a grade 1 open fracture (4%), both AO/OTA 12A1. Two periprosthetic fractures were included with a fixed stem and four peri-implant fractures (13%): three around a humeral plate and one around an intramedullary nail. Four fractures (8%) were pathological, one was due to an underlying bone cyst, two were due to metastases, and one due to Paget disease. There was one infected nonunion case that occurred after intramedullary nailing performed five months earlier. In three cases, the RNBP was used as replacement of early failed osteosynthesis, one IMN, one helical plate and one double plate osteosynthesis distal from a shoulder prosthesis. Seven patients suffered from a primary RNP after a fracture.

### Surgical details

Surgical details are presented in Table 1. The mean duration of primary surgery was 116 min (range: 49–203) for 46 of the 48 patients. The time of surgery for two patients could not be retrieved since they underwent additional surgical procedures during the same session. Twenty-one patients (44%) underwent lateral plate fixation using a direct lateral approach. Nine cases involved proximal one-third shaft fractures, nine cases involved middle one-third shaft fractures, and three cases involved distal one-third shaft fractures. The posterior approach was applied in 27 patients (56%). Of these patients, two had a proximal one-third shaft fracture, 13 had a middle one-third shaft fractures, and twelve had a distal one-third shaft fracture. The posterior approach group included the only nonunion case in the cohort. This case involved replacing the IM nail with a posterior RNBP. The only helical plate in the cohort was applied posteriorly. A compression technique was applied in 35 fractures (73%), a bridging technique in 10 fractures (21%) and in three fractures (6%) a mixture of principles was applied. Of the three peri-implant fractures involving plates, the plates were left in place in two cases and in one case the two supracondylar plates were replaced with longer plates. In the peri-implant fracture case involving a nail, the nail was replaced with a lateral RNBP.

### Implant removal

Eleven patients (23%) underwent RNBP removal, details are presented in Table 2. All plate removals except one were performed by the same orthopedic surgeon who had performed the original RNBP procedure. None of the patients had an iRNP, but one patient experienced a 3 day weakness of the index finger extension, which occurred after cauterization used at the level of the wave. In one case the removal was performed at the patient’s request.

**Table 2:** Patient characteristics of cases with plate removal

	RNBP removal (*N* = 11)
Age (years)	63 (29–77)
Sex (female)	9
Reason plate removal	
Nuisance/pain	9^*^
New fracture	1
Nonunion	1
Plate position	
Lateral	7
Posterior	4
Primary RNP	0
iRNP after primary surgery	2
Interval (months)	25.5
Surgery duration (min)^**^	48 (35–65)
iRNP after removal	0

Data are presented as mean (range) or N

^*^In one case a nonunion was diagnosed after plate removal

^**^Data was available for 6 cases

iRNP, iatrogenic radial nerve palsy; RNBP, radial nerve bridging plate

Three cases were due to residual symptoms at the shoulder level. Four were removed due to diffuse residual symptoms in the upper arm likely linked to the implant’s presence. The plate was replaced in three cases: two due to nonunion and one due to a new, post-traumatic, peri-implant fracture proximal to a lateral RNBP that had been applied eight months prior. One plate was removed elsewhere. In this case the procedure took 160 min, since the operating surgeon did not realize that the radial nerve was under the plate.

### Complications

Of the 41 patients who had no primary RNP after the fracture, four patients treated with a lateral approach and three with a posterior approach experienced a temporary iRNP after primary surgery. The mean recovery time was 12.3 weeks (range: 1–32). No malunions occurred, three patients (6%) had a malalignment between 10 and 15° in the profile view and one (2%) between 10 and 15° in the anteroposterior view. All four belonged to the bridging group. There were three nonunions (6%) and one (2%) early failure of osteosynthesis. In three patients (6%) a breakage of the locking screws occurred. One patient (2%) experienced a deep infection after a posterior RNBP, which was diagnosed one week after surgery. Details of these complications can be found in Table 3.

**Table 3:** Complications

Complication	AO-OTA	Fracture location	Approach	Technique	Treatment
Nonunion^1,2^	12C3	Middle third	Lateral	Mixed	RNBP was replaced by IMN
Nonunion^3^	12A1*	Proximal third	Lateral	Bridging	IMN was added
Nonunion	N.A.	Distal third	Lateral	Compression	Treatment of surgical neck nonunion with plate osteosynthesis
Implant failure^2^	12C3	Proximal third	Lateral	Bridging	Revision of secondary displacement with retention of the RNBP
Deep infection	12B2	Middle third	Posterior	Compression	Daily wound cleaning and antibiotics
Malalignment^2^	12B3	Proximal third	Lateral	Bridging	Expectative
Malalignment	12B2	Proximal third	Lateral	Bridging	Expectative
Malalignment	12A3	Middle third	Posterior	Bridging	Expectative
Screw breakage	12C3	Proximal third	Lateral	Compression	Expectative
Screw breakage	12A1*	Distal third	Posterior	Compression	Expectative
iRNP	12A1	Middle third	Lateral	Compression	Expectative
iRNP	12C2	Distal third	Posterior	Compression	Expectative
iRNP	12A2	Middle third	Posterior	Compression	Expectative
iRNP	12B2	Middle third	Posterior	Bridging	Expectative

1 This patient had also screw breakage

2 This patient had also iRNP

3 This patient had also malalignment

*Pathological fracture

IMN, intramedullary nail; iRNP, iatrogenic radial nerve palsy; RNBP, radial nerve bridging plate

## Discussion

Data from the current study demonstrated that removal of a RNBP is straightforward and safe, as none of the 11 patients experienced an iRNP. The RNBP technique has a high union rate of 94%. There were three nonunions (6%), one of which was a pathological fracture at the humeral shaft and one nonunion of a surgical neck fracture. Seven patients (17%) experienced a temporary iRNP after primary RNBP surgery.

The use of a wave plate has previously been described for the treatment of femoral nonunions [16]. The wave was created at the level of the nonunion to avoid bending forces at the level of the plate. Introducing a wave into a straight plate did not weaken the plate significantly regardless of the length or height of the wave [18, 19]. Although the biomechanics of a femoral wave plate are different to those of a humeral RNB plate, these studies show that, even in weight-bearing plates, bending does not result in plate weakening. In the current study, no postoperative plate breakage or secondary plate bending was observed, suggesting that the RNBP maintained its strength. One possible disadvantage of the wave is that screw fixation is not possible at the level of the wave section due to the presence of the radial nerve. This may theoretically interfere with the stability of the construct, especially in cases where the fracture is located at the level of the wave, as with posterior plating. However, this was not identified as a problem as there were no cases of nonunion in posterior plating.

The incidence of iRNP after treatment with a RNBP was likely due to the learning curve associated with safely exposing the radial nerve and seemed not to be related to the RNBP technique itself. This can be inferred from the fact that six out of seven iRNP events occurred during the first three years of the 10.5-year study period, during which 22 out of 41 cases were treated. These seven cases of iRNP occurred in four of 21 patients treated using a lateral approach (19%) and in three of 27 patients treated using a posterior approach (11%). Because the radial nerve had been explored during the primary procedure and was known to lie safely beneath the wave segment of the plate, a wait-and-see approach could be adopted, resulting in complete recovery in all iRNP cases. Keeping the radial nerve in its natural anatomical bed, rather than stretching it over the plate, may influence both the incidence of iRNP and the time required for recovery of a RNP. This is supported by studies demonstrating better pRNP recovery outcomes with plating techniques that avoid direct plate–radial nerve contact (e.g. anteromedial plating) compared with techniques in which the radial nerve lies in contact with the plate (e.g. posterior plating). Gunsoy et al. reported that in humeral fracture plating with concomitant primary RNP, anteromedial plating was associated with significantly faster nerve recovery (23.9 ± 4.0 weeks) compared to the posterior approach (35.0 ± 8.2 weeks) [20]. In cases of iatrogenic radial nerve palsy (iRNP), a meta-analysis by Shon et al. of nine studies suggested lower recovery rates with posterior plating versus anterolateral plating; however, the difference was not statistically significant [21]. All patients had complete recovery of nerve function with recovery times being shorter for iRNP than for primary RNP (a mean of 12.3 weeks vs. 23.4 weeks) which is consistent with the literature [3, 22]. This duration is shorter than the reported recovery time of pRNP after anteromedial plating (23.9 weeks) and posterior plating (35 weeks) [20]. Based on this difference in recovery time, Gunsoy et al. suggested that having no contact between the nerve and the plate could be a reason for the relatively short recovery time.

A non-union rate of 6% (3 of 48 cases) is consistent with the literature, where nonunion rates range from 3% to 6% in patients treated with plate fixation [6, 23]. However, among those three cases, one was a nonunion of a pathological fracture in which fracture healing is severely compromised and one was a nonunion of the surgical neck component of a multifocal humeral shaft fracture with a healed shaft component. Additionally, all nonunions occurred in the lateral RNBP group, a technique the author has replaced with helical plating since 2017. This technique appears to be most beneficial in posterior plate fixation as there were no patients with nonunion in this group. As the anterolateral approach no longer requires bridging the radial nerve, the RNPB technique is particularly advantageous for fractures of the mid and distal third of the humerus treated with posterior plating or in cases where RN exploration seems indicated for reasons relating to direct reduction techniques or osteosynthesis techniques associated with an increased risk of radial nerve injury (e.g., cerclage wiring), or in primary RNP cases.

The reintervention rate of 25% (12 out of 48 cases) was higher than typically reported in the literature. According to a meta-analysis that included 28 studies with 5,490 patients and compared plate and nail fixation for humeral shaft fractures, the reintervention rate for reasons of iRNP, infection, nonunion, or implant failure was 12.6% after open plate fixation [6]. However, discomfort was not cited as a reason for plate removal. Similarly, in another meta-analysis comparing nonoperative and operative treatment of humeral shaft fractures, including 12 studies with 1,412 patients, plate irritation was cited as a reason for removal in only one study [24]. Implant removal is frequently offered as a solution for residual symptoms after fracture healing [25]. Reasons for implant removal after fracture healing in upper and lower limbs were related to the patient’s wishes in 51.1% of cases, pain in 30%, and mechanical irritation in 17% [26]. Although implant removal due to discomfort may significantly reduce complaints, the low rate of plate removal in healed humeral fractures can be explained by the reluctance of most orthopedic surgeons to remove humeral plates in order to avoid damaging the radial nerve [26, 27]. In a study comparing the anterolateral and posterior approaches in the treatment of humeral shaft fractures, implant removal for pain relief was mentioned in 10.0% of patients in the anterolateral group and 12.3% in the posterior group [28]. This study stated that implant removal was more challenging in the posterior group due to scar tissue formation around the radial nerve. However, no data were provided regarding complications associated with plate removal. Another study, reporting outcomes after an anterior humeral approach for distal shaft fracture of the humerus in young patients, reported implant removal in 24 of 31 (77.4%) cases [29]. Implant removal was performed at the patient’s request in 17 cases, to improve elbow flexion in two cases, and relief of discomfort in five cases. Clearly, as reflected in the current series, the RNBP technique significantly lowers the threshold for removal for any reason. However, it remains unclear whether the RNBP itself increases the risk of discomfort compared to standard plates. When excluding plate removal due to discomfort or in the one asymptomatic patient, four reinterventions remain in this cohort: three for nonunion and one for early failure.

This study has some limitations. It is a relatively small, retrospective, single-surgeon study, and its results may not be generalizable to all surgeons. The cohort was rather heterogeneous including primary fractures, as well as nonunion and peri-implant fractures. Additionally, this study lacks a comparison group, an attempt was made to compare the current rate of iRNP in case of plate removal with recent literature. However, the literature does not provide specific data on incidence, though iRNP appears to be at least as prevalent as after primary plating. It may therefore be valuable to compare the findings of the current study with a cohort treated using a traditionally placed humeral plate over the same timeframe. A direct comparison with such a cohort was not feasible, as the RNBP was used whenever indicated, leaving very few patients with a conventional plate at this single center. Accordingly, this study’s findings are interpreted solely in the context of existing literature. Consequently, it remains unclear whether the RNBP technique reduces iRNP after plate removal. Bending the plate is a trial-and-error process that requires adaptation to each patient’s anatomy. Although the rates of malalignment and nonunion are consistent with those reported in the literature, it cannot be definitively concluded that these outcomes are unrelated to the wave plate technique itself. The absence of a standardized bent plate design or a precontoured template limits the ability to fully evaluate plate-related risk factors that might become apparent in a larger study.

## Conclusion

The radial nerve bridging plate technique prevents radial nerve palsy when the plate is removed while maintaining the effectiveness of plate fixation for treatment of a humeral shaft fracture. The RNBP technique facilitates the decision to remove an implant if a patient experiences persistent symptoms or desires removal of a plate.

## Data Availability

Data are available upon reasonable request. Proposals should be directed to the corresponding author.
